# Objective computorized evaluation of normal patterns of facial muscles contraction

**DOI:** 10.1590/S1808-86942012000200008

**Published:** 2015-10-20

**Authors:** Jose Jarjura Jorge, Paulo Roberto Pialarissi, Godofredo Campos Borges, Sara Agueda Fuenzalida Squella, Maria de Fatiima de Gouveia, Jose Carlos Saragiotto, Victor Ribeiro Gonçalves

**Affiliations:** a PhD. Full Professor – Medical School – PUC – SP. Coordinator of the ENT Program – PUC-SP; b PhD. Full Professor – Coordinator of the Biomedical Engineering Program – PUC-SP); c PhD. Associate Professor – ENT Program; and Head of the Surgery Department – PUC-SP); d PhD. Scholarship Holder – Renato Archer Informaton Technology Center; e PhD – Renato Archer Informaton Technology Center; f Student – Biomedical Engineering Program – PUC-SP; g Student – Biomedical Engineering Program – PUC-SP. Pontfcia Universidade Católica de São Paulo: Faculdades de Medicina e Ciências da Saúde e Faculdade de Engenharia Biomédica

**Keywords:** facial expression, facial muscles, facial nerve, facial paralysis, movement disorders

## Abstract

Different methods used to evaluate the movements of the face have many degrees of subjectivity and reliability. The authors discuss the ease of using these methods in clinical practice or their accuracy in scientific research.

**Aim:**

To obtain the standard for normal facial muscles movements using an objective method – the Vicon system.

**Materials and Methods:**

Light reflective markers were placed at points of interest on the face of 12 normal subjects. The movements were captured by cameras that sent the images to a computer. The points' displacements were measured between rest and maximum muscle contraction; and we calculated the means and the standard deviations (SD) were calculated.

**Results:**

When smiling, the variation of the oral commissures was between 6.45 and 12.11 mm, mean of 9.28 mm and SD od 2.83; for lifting the eyebrow, it is between 6.0 and 13.08 mm, mean of 10.57 mm and SD of 2.51; for eyelids movement there was a variation of 6.89 and 11.29 mm, with a mean value of 9.09 mm and SD of 2.20; for the movement of wrinkling the forehead, the results showed a variation of 4.16 and 10.85 mm, with a mean value of 7.56 and SD of 3.29.

**Conclusion:**

The authors obtained normal patterns for facial muscle contraction.

## INTRODUCTION

The face reveals our innermost expressions, it is an essential part of human communication and its traces mark our individuality^1^. This happens by means of the fine control over facial muscle contraction, either voluntary or involuntary, carried out by the Facial Nerve or VII cranial nerve.

Peripheral Facial Paralysis (PFP) may change the individual's mood, changing facial movements and thus the facial appearance. It may bring about changes to the individual's appearance and thus in his/her social behavior, causing loss in work and in social living^2^.

Facial paresis or paralysis are symptoms which indicate facial nerve disorders, which may be caused by trauma, infection or tumors. The VII cranial nerve, which according to Santos-Lasaosa^3^, is the most commonly paralyzed nerve in the human body. Besides being responsible for facial expression, it is also responsible for tearing, salivation and taste; and, because of facial muscle laxity, usually unilateral, not only the facial movement is impaired, but also the patient will have difficulties to speak and feed. The main sequela may involve the eyes, because of lack of eyelid closure, it may cause corneal ulcers and the consequent damages to it.

Individuals vary in terms of their facial expressions; there are also differences between the two facial sides, gender and age^4^ differences as well, which makes it difficult to standardize the measure of movements.

For this reason, throughout the history of PFP treatment, physicians have tried to grade the paralysis in order to quantify and report it. Since 1955, numerous methods and scales have been created in order to assess with more or less accuracy the facial movements and to quantify the disorders which affect such movements.

We may break them down into subjective; objective; simple methods which use regular rulers and photographs; and complex methods, using fancy equipment with digital cameras, computers, software, scanners and lasers.

The first grading system was introduced by Botman & Jongkees, in 1955. It was based on a simple scale with five categories, from 0 (normal) to IV (complete paralysis). Nonetheless, until 1983, we did not have a standard to compare with and check whether there had been an improvement or not of the facial paralysis after clinical or surgical treatments^5^. In 1983, House introduced his 6-category scale; and in 1984 Brackmann & Barrs also published a grading system used to analyze the differences between the normal and the affected sides. In 1985, The Facial Nerve Disorders Committee of the American Academy of Otorhinolaryngology and Head and Neck Surgery adopted, as universal standard, a grading system based on the studies carried out by House and Brackmann, which is used all over the world today and by all researchers in the field^6^. Many other scales have been suggested, for instance, the one from Yanagihara, used in Japan, which assesses 10 facial areas; but it does not consider secondary changes, such as synkinesia^7^.

Burres & Fish^8^ introduced an objective method to measure distances between specific facial points during rest and five standard movements comparing the affected side on the face with the normal one (Burres and Fish's Linear Measure Index), using photographs and video. In 1994, Murty et al., simplified the system, calling it the Nottingham System, preserving the three facial expression measures, having them in percentages and specifying whether secondary effects were present or not, and if the patient had tearing, dry eyes and taste changes.

Fields & Peckitt^9^ described a simple and practical method: one measure is done with the mouth closed and at rest, from the corner of the lip to the external corner of the ipsilateral eye. Afterwards, the patient is asked to smile broadly and a new measure is taken. These numbers are placed in a formula and the result is called Facial Nerve Functional Index, expressed in percentage.

Ross et al.^10^ developed a grading system which was called Toronto Facial Grading System, which scores the facial muscle movements from 100 (normal) to 0 (totally paralyzed). They considered the assessment of the compromised side at rest compared to the normal side; the difference of maximum movement at the involved side compared with the normal side and the degree of synkinesia.

Coulson et al.,^11^ analyzed the numerous existing objective methods, and they concluded that those that assess bidimensional movements are not appropriate, because facial muscle movements happen in the anteroposterior, horizontal and vertical axis. Thus, the tridimensional analysis would be more precise. They used 19 spherical marks in strategic points on the face and four video-cameras with reflectors. They captured the images and processed them using the “Expert Vision Motion Analysis System” and the Miniplan statistical analysis software.

Isono et al.^12^ used a similar system of reflective marks on the face at the highest points and at the median points of the eyebrow, bilaterally, on the nose base and tip; introducing, however, one camera with infrared light flash (Qualisys) and one video processor with the Excel (Microsoft) software and Wingx (Informix) in order to statistically analyze the movements.

The Moiré Topography method is an optic measure which enables the visualization of the facial contour in three dimensions, creating stria as a map of contours with a high degree of accuracy. It is obtained using a Moiré irradiation camera (Fuji 3013), coupled to a TV camera and a video-monitor. In 1997, Yuen et al.^13^, studied three regions where the number of stria created by the method are counted. The number of stria in the paralyzed side is then divided by the number of stria in the normal side, divided by one hundred, creating the: Nasolabial Groove Moiré Index; Oral Angle Moiré Index and the Internal Eye Corner Moiré Index. They compared it to the HB scale and found a high correlation, especially in the nasolabial groove and mouth angle indices.

Bajaj-Luthra et al.^14^ quantified the facial synkinesia by means of the Maximal Static Response Assay, a technique described by Johnson et al.^15^, which enables the quantitative assessment of facial movements with the use of a method to place 1mm adhesives in pre-defined points on the face: the glabella, the upper lip groove, the chin, supraorbital, infraorbital and in both corners of the mouth. In order to calibrate the distances, they used a 2cm ruler on the nasal tip. For each movement, each patient is asked to do a maximum contraction movement and to keep the maximum shifting position. Following, the movements are filmed, raising the eyebrows, closing the eyelids, forced smile, and others. Then the images are captured by a Targa+ panel and one Jandel Scientific Sigma Image software. They are all calibrated at a common origin by means of a system of Cartesian coordinates.

Frey et al.^16^ reported that special and sophisticated methods are necessary in order to measure facial movements in cases of motor deficiencies and their recovery. They used the two-mirror system in a 90-degree angle and a camera to capture different sides of the face at the same time in a video screen. After filming the movements, they are analyzed by a software which measures the distances and the tridimensional movements of the frontal images and those of the right and left side images. In the beginning, they used the VICON system for a tridimensional analysis of the movements, which they considered very useful for scientific purposes, though more complicated for the day-to-day clinical practice. They, then developed what they call the FACIOMETER, a digital metering device which measures the distances between facial movement points. They are established and marked with non-washable ink, with static points: nasal tip and tragus; and also the dynamic points to be studied: eyebrow, lower and upper eyelids, nasal wings, upper labial sulcus, mouth corner and mid-lateral points on the upper and lower lips. Then, the individual is filmed and asked to do the maximum movements of raising the eyebrows, close the eyes, showing the teeth, smiling and whistling.

Cohn et al.^17^ created a facial image analysis system to detect, extract and recognize emotion expressions which have been used to show subtle changes in facial movements after interventions. This system requires 40 points, using video images and a computer.

In the same year, O'Grady et al.^18^, made the techniques even more sophisticated by using the third dimension (3D) and the Laser beam in order to measure the facial movement. With the Laser, the individual's face is scanned and two digital cameras capture the signal which, by triangulation, provides the measure of depth. The image is processed by a software (Cyberware Echo). The authors used plastic anatomical models of the human face, of natural size and color. One perfectly symmetrical and another with facial asymmetry.

Kayhan et al.^19^ utilized the Toronto Facial Grading System, described by Ross in 1996, to assess patients with facial neuromuscular involvement and to measure the method's sensitivity among five different observers who analyzed the patients' videos and assigned a grade to each movement. They concluded that the method is good and that there was, statistically, an agreement among the observers.

In the year 2000, Linstrom et al.^20^, used a new, commercially available, interactive video system, the “The Peak Motus Motion Measurement System”, which objectively measures the asymmetries of selected points on the face during eye closure and smiles. The fixed points are marked with a 3M reflexive tape on the nasal dorsum, right and left upper eyelid, right and left labial commissure. The images were captured by a camera (Sony CCD-TR56) in black and White and recorded in a videotape and, later on, sent to a computer to be processed by image edition software: Super Mac Screen Play, Adobe Premier and the NIH image, NIPG Dip Statin. The reference point to calculate the shifting of the other points was the glabella. In normal individuals, the mean smile variation in the greater axis was 0.578 cm with an SD of 0.209 to the right and 0.553 cm with an SD of 0.183 to the left and, for eyelid closure it was 0.811 cm with an SD of 0.239 to the right, and 0.794 cm with an SD of 0.210 to the left. In 2002^21^, they used the same system and analyzed two movements: eyelid closure and smile in normal individuals and patients with facial paralysis after surgical treatment of acoustic neuroma. The point of reference for the calculation of shifting of the other points was the glabella. In normal individuals, the mean variation in smile, in the larger axis was 0.578 cm with an SD of 0.209 to the right and 0.553 cm with SD of 0.183 to the left and; for eyelid closure, it was 0.811 cm with an SD of 0.239 to the right and 0.794 cm with an SD of 0.210 to the left. The statistical analyses showed that there was no significant difference between the right and left side.

In 2001, Wachtman et al. described^22^ a new method, the FAA – Facial Automated Analysis, and compared it with the Maximum Static Response Analysis (MSRA). In the FAA it is not necessary to place the markers on the face and they used computer programs to extract and quantify the facial dense flow extraction movement, facial tracking and high gradient component detection.

Hontanilla & Auba^23^ presented a tridimensional method to capture facial movements, the Facial Clima, which is a system of automatic optical movements which involve the placement of reflective points on the face, filmed with three infrared cameras. The images are processed by a software which processes distances and, which the authors consider different from other methods, besides the advantage over them: the measure of the velocity of the areas studied.

In 2008, Mitre et al.^24^, used a photographic method with fixed points on the face and compared them to the HB scale. They photographed normal individuals with PFP on the following situations: at rest, raised eyebrows, closed eyes, smiling and whistling. The photographs were scanned and analyzed with the Corel Draw Work Tools (version 9). They obtained the results which were expressed in percentages, the lowest value divided by the highest and multiplied by one hundred. Thus, they called it the Facial Asymmetry Index (FAI). Comparing with the HB classification, they arrived at the following conclusion: HB = I – FAI = 97.1 to 100%; HB = II – FAI = 93.1 to 97.0%; HB = III – FAI = 91.1 to 93.0%; HB = IV – FAI = 88.1 to 91.0%; HB = V – FAI = 84.1 to 88.0%; HB = VI – FAI = 84.0% or less.

Also, in 2008, Manktelow et al.^25^ developed an easy and cheap way to measure facial movements using two conventional transparent rulers placed in the horizontal and vertical directions and a marker, measuring only the mouth slit movement, during rest and smiling. In average, they obtained the following values for labial commissure movement: from 7 to 22 mm, with a mean value of 14 mm, and for the upper lip medium point movement it was between 5 and 19 mm, with a mean value of 11 mm. The authors submitted the patients to two examiners, who carried out three measures for each movement, using the data from the largest movements measured. The statistical calculations between the results of the two examiners who showed reliability and method precision.

Quintal et al.^26^ used a digital pachymeter to determine the normality pattern for the differences measured between the hemifaces of normal individual and the patients with peripheral facial paralysis. They found differences between the measures of the labial commissure and tragus of normal individuals, of 3.26 mm; from the eye internal corner and the labial commissure, 0.83mm; between the external eye corner and the labial commissure, 2.45 mm in smiling and 4.88 mm in nasal contraction.

The various methods described vary in different degrees of subjectivity and, thus, their reliability is discussed. We also discuss the practicality of the method to be utilized in the daily clinic or the precision to be used in scientific papers.

There are a lot of studies in the field in order to enhance the systems. One ideal system would be one of easy handling, low cost, requiring minimum equipment and little time for its performance, capable of measuring the static and dynamic functions of the facial muscles^5^.

This study aims at assessing an objective method to measure facial muscle contractions, in order to obtain a normality pattern so that later we can use it to compare cases of facial paralysis.

## MATERIALS AND METHODS

Vicon is an image capture system made up of cameras with LEDs. They are capable of capturing each positioning of the reflectors in space.

The reflexive points are positioned on the individual, in the region where one wants to study a given movement, such as, gait, in such a way as to involve the upper and lower limbs, trunk and head.

**Figure 1:**
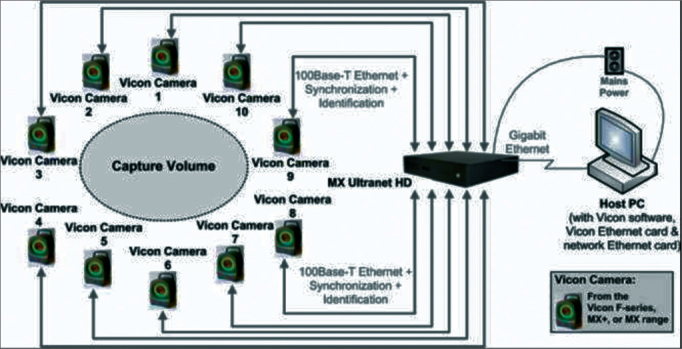
Scheme of how the Vicon capture system works.

The system is made up by:

• T160 cameras, with 16 MP capacity, infrared images of up to 100 frames per second. The camera structure emits infrared light which, when reflected, is captured by the camera itself.
• The Giganet unit connects all the cameras to the control computer.
• Circular retro reflector markers may be placed in the individuals to track their movements in three dimensions.
• The software utilized is the Vicon Nexus, which provides a real time vision of the movements of the markers in three dimensions and it also has a capacity to store a large amount of data.
• The T-shaped calibration rod, with four retroreflective markers at a given distance, is used to calibrate the camera positions with the software. There are also two indicators to place the rod horizontally on the floor and observe its location in space.
• The control computer translates the information supplied by the Giganet Unit by means of the Nexus software in a three-dimensional image. It enables the linear and angular velocity calculations of the joints, common centers and the mass segment centers. Normally utilized to analyze the dynamic gait, activities and postural stability^27^ (Figure 1). The tests were carried out in the ICU.

**Figure 2:**
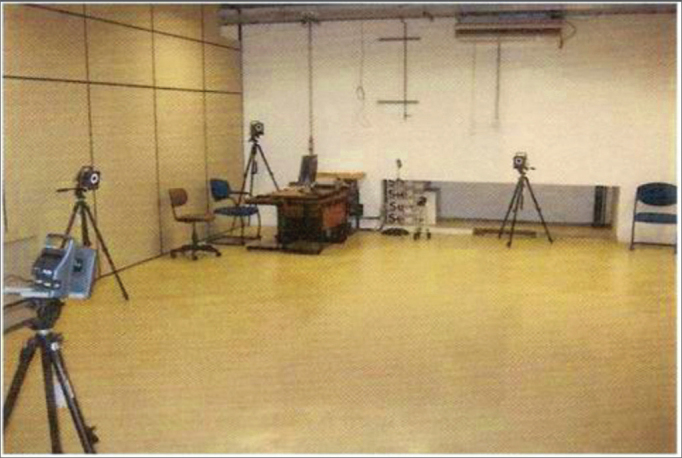
Movement capture room.

We used a movement capture room (Figure 2). The cameras were placed around the capture space in such a way that there were no hidden markers, because of lack of coverage, by at least two cameras.

*For the present study, we used only three cameras, enough to capture the desired points in the study.*

### The new markers

The Vicon systems usually supplies markers of two sizes, in the shape of a hemisphere of 14.5 and 9.5 mm which are not good for light reflection in small areas such as the face and the eyelids. Thus, new markers with 4mm diameters coated with reflective tape, were created in the ICU laboratory, with the help of a microscope.

### Procedures

#### Markers

There is no facial model included in the software; thus, the need to create a new one. The person who performs the test is responsible for defining the points which better serves his/her study.

**Figure 3:**
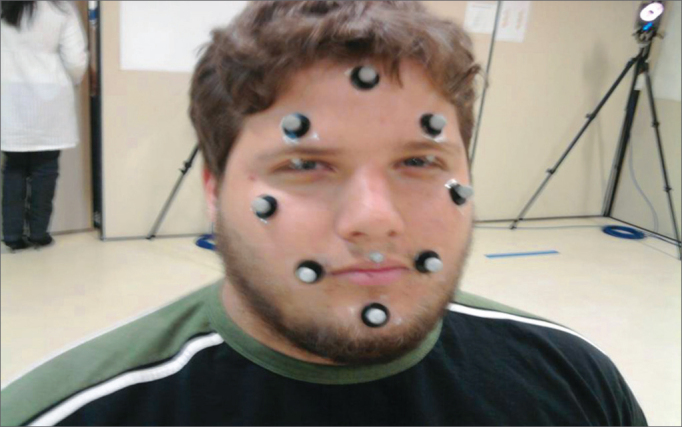
Examining the markers established at strategic points on the face.

We defined 11 markers in strategic points on the face (forehead center near the hair line, central position above both the eyebrows, malar regions in the line of the eyes external corners, center of both upper eyelids, corners of the labial commissures and chin); and we noticed, on the image produced in the monitor by the software, that these points were sufficient and satisfactory for the study's objective (Figure 3).

**Figure 4:**
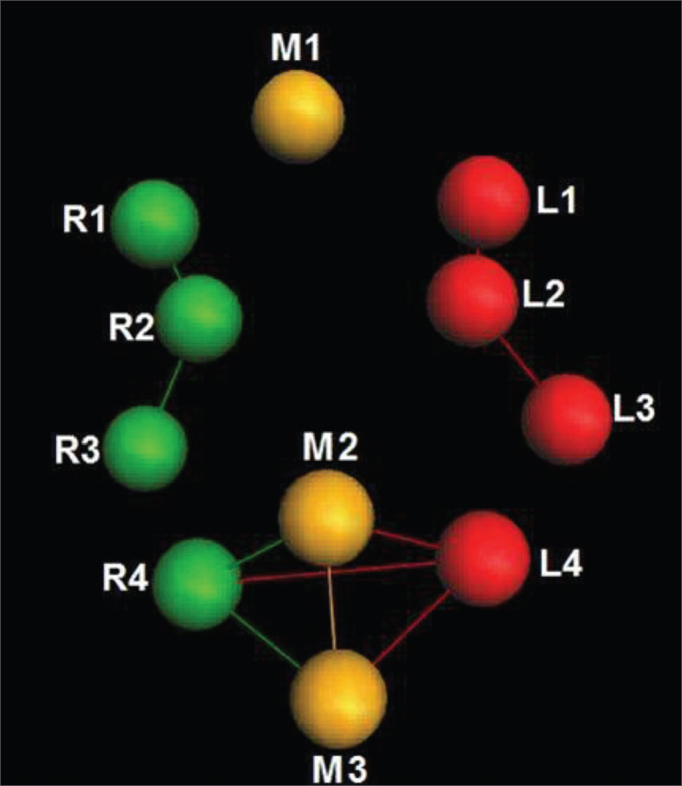
Markers established with its respective names.

Each marker utilized is represented by a specific name that the very software establishes (Figure 4).

The markers were stuck to the skin of individuals after the use of a degreasing substance. The individuals tested were placed at a strategic point in the room, where the cameras captured the markers movements. One static model of the individuals was captured to be recognized during the other stages of the capture process.

#### Camera calibration

Before capturing any image, the cameras must be synchronized in a calibration process. Upon shaking a rod through space, where the movements must be captured, the software calculates the relative position of the cameras. The rod was placed in the indicators and the software established the floor level. In this process, the virtual space is equal to the real space. After calibration, only two cameras are needed to identify the location of a marker up to 0.1 mm of accuracy. Nonetheless, as we said before, three cameras were used for this study.

We carried out the tests in 12 normal individuals, from both genders, with ages between 18 and 21 years, after signing the consent form. Each individual was asked to perform the following movements: smile, wrinkle the forehead, blink the eye and frown.

The images captured were sent to a computer, processed and analyzed through the Vicon Nexus software.

The tests were carried out and, for our study, we assessed the variation of the point strategically placed on the face when the facial muscles were contracted by the individual, so that, based on this, we can reach a facial contraction normality pattern.

**Figure 5:**
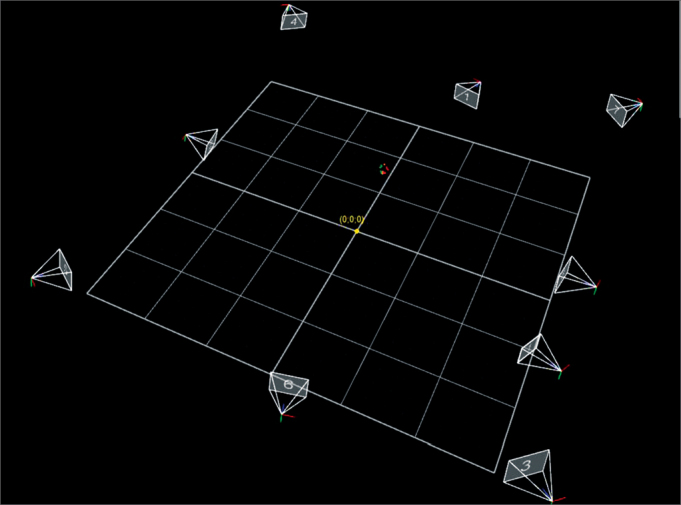
Showing the origin (0 0,0).

The Vicon Nexus software measures the shift from the point when the facial muscles are contracted. The software captures the shifting from the point on axis x, y and z, always in relation to an origin (0 0,0) (Figure 5).

**Figure 6:**
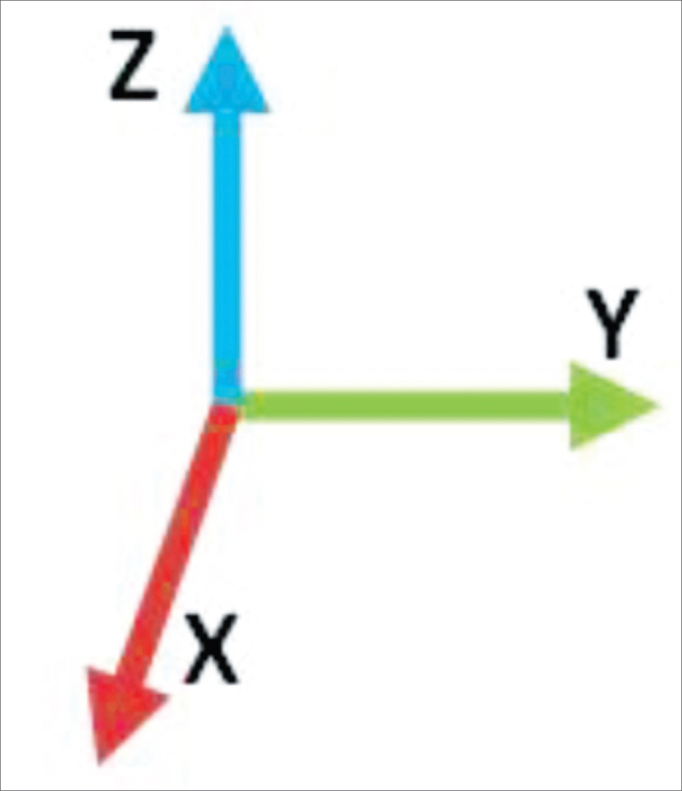
X, Y and Z Axis.

We observed that the axis shows a larger variation and what interests us is the z, because in this axis the facial muscles presented a variation pattern when contracted (Figure 6).

Each movement had only two markers selected to be studied, because these two markers were already enough to obtain a measure pattern. The other markers were not considered in this study.

The data obtained from each individual were plotted and each case was worked and studied, generating an average value for each facial movement.

## RESULTS

Following, the obtained results. In each movement of the chosen axis, we chose the point at rest and the maximum contraction point in the movement. The numbers obtained and recorded in the charts are marked in relation to the point of reference.

### Smile

**Chart 1:**
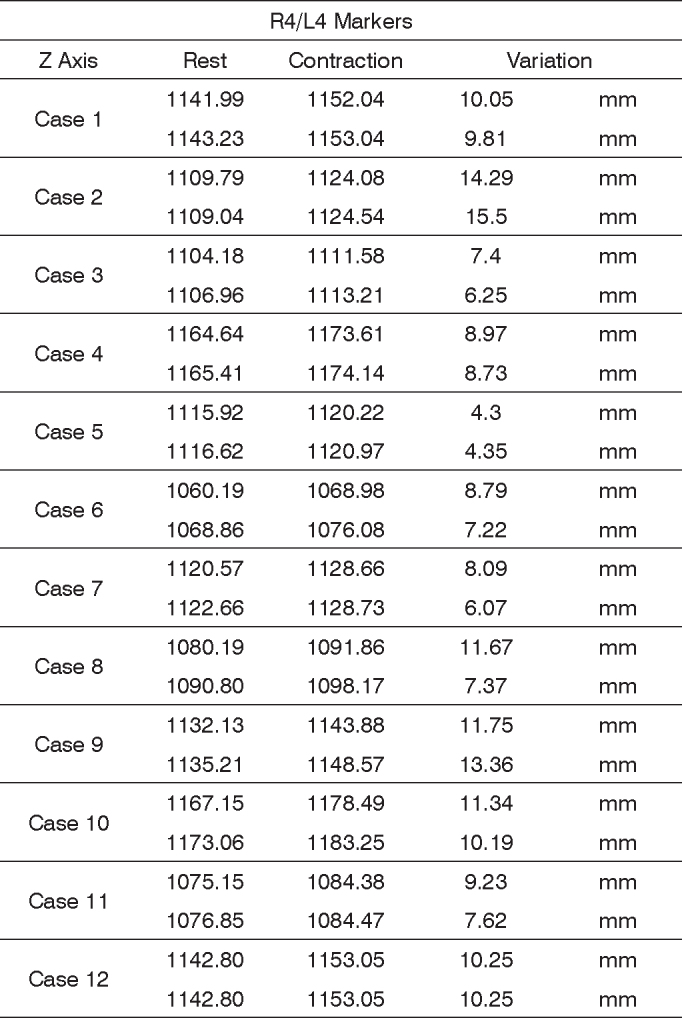
Values obtained in millimeters (mm), according to the reference point, in smile, at rest and contracted in the Z axis.

In order to analyze the movements upon smiling, for each case we used the R4 and L4 markers, being symmetrical. The results are plotted on Chart 1. Based on data analysis, we concluded that, in a normal individual, the variation upon smiling must be of 6.45 mm to 12.11 mm on the Z axis.

**Figure 7:**
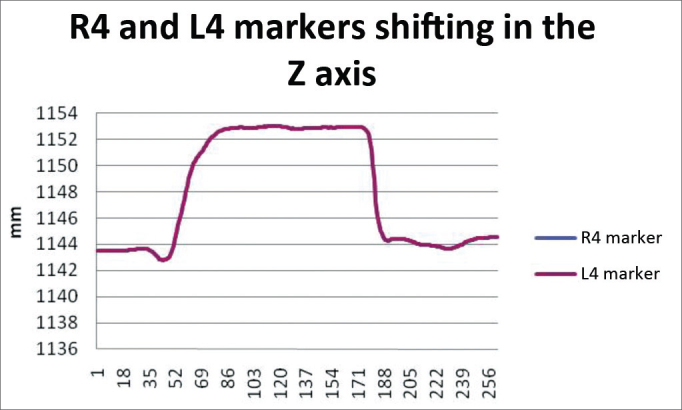
R4 and L4 markers shifting in the Z axis, smiling.

We obtained a 9.28 mm variation mean value, with a variation standard deviation of 2.83 mm (Figure 7).

### To wrinkle the forehead

**Chart 2:**
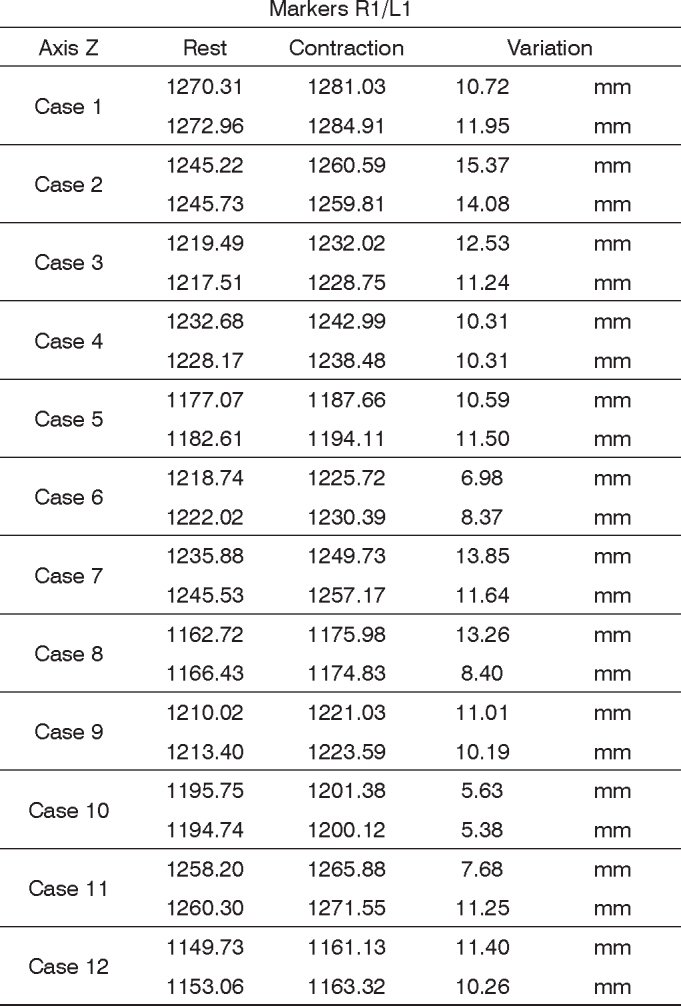
Values obtained in millimeters (mm), according to the reference point, upon frowning, at rest and contracted at the Z axis.

In order to analyze the movements needed to wrinkle the forehead, for each case we used markers R1 and L1; both being symmetrical. The results are plotted on Chart 2. Based on data analysis, it was concluded that in a normal individual, the variation upon wrinkling the forehead is 8.06 mm to 13.08 mm on the Z axis.

**Figure 8:**
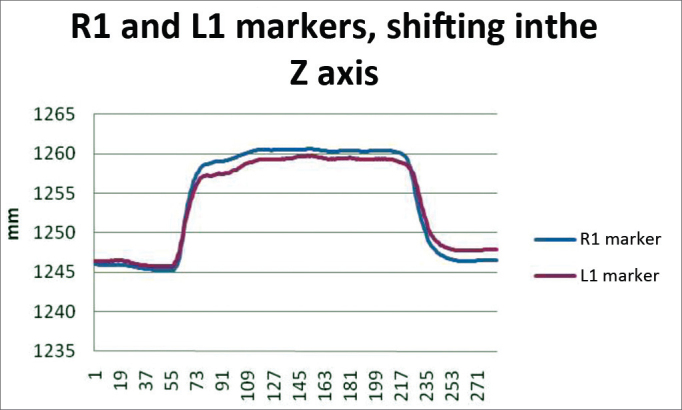
R1 and L1 markers, shifting inthe Z axis, frowning the forehead.

We obtained a variation mean value of 10.57 mm, with a variation standard deviation of 2.51 mm (Figure 8).

### Close the eyes

**Chart 3:**
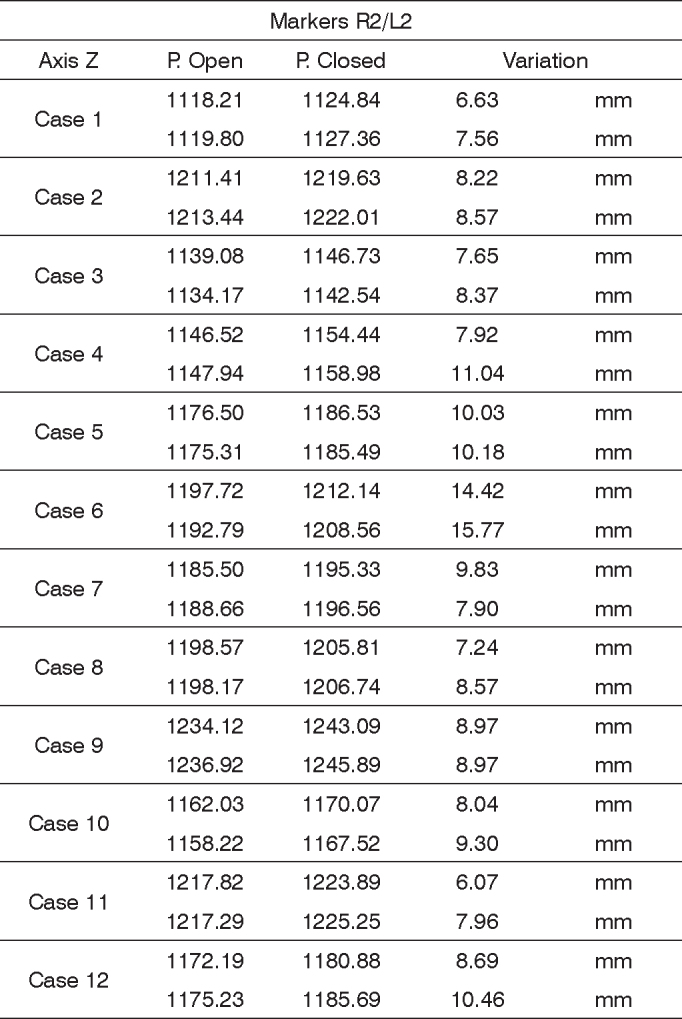
Values obtained in millimeters (mm), according to the point of reference, in closing the eyes at the Z axis. P = eyelid.

In order to analyze the movements for closing the eyes, for each case we used markers R2 and L2, being both symmetrical. The results are plotted on Chart 3. Based on the data analysis, we concluded that in a normal individual, the variation upon closing the eyes must be of 6.89 mm and 11.29 mm on the Z axis.

**Figure 9:**
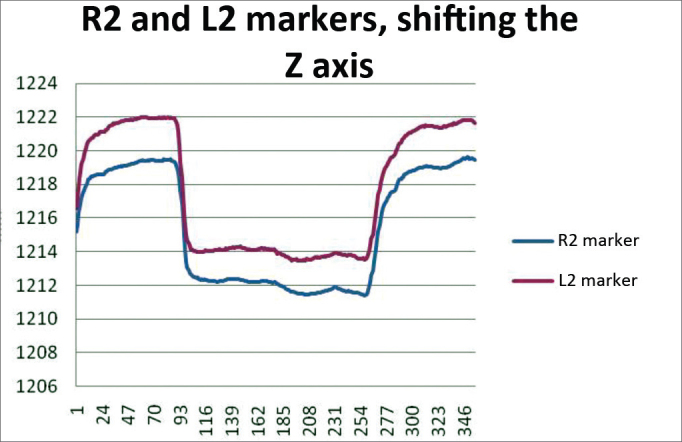
R2 and L2 markers, shifting the Z axis, closing the eyes.

We obtained a variation mean value of 9.09 mm, with a variation standard deviation of 2.20 mm (Figure 9).

### Frowning

**Chart 4:**
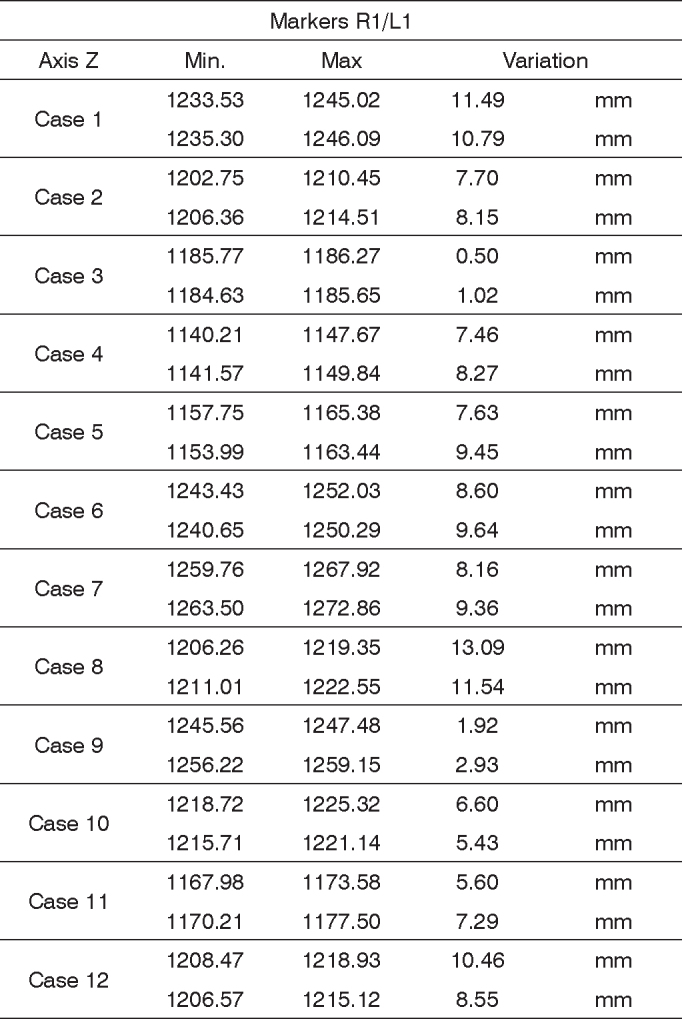
Values obtained in millimeters (mm), according to the reference point, upon frowning, at rest and contracted in the Z axis.

In order to analyze the frowning movements, for each case we used the R1 and L1 markers, them being symmetrical. The results are plotted on Chart 4. Based on the data analysis, we concluded that, in a normal individual, the frowning variation should be between 4.26 mm and 10.85 mm on the Z axis.

**Figure 10:**
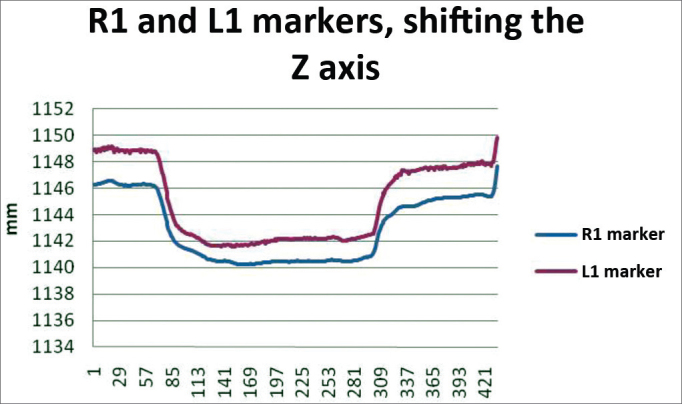
R1 and L1 markers, shifting the Z axis, frowning.

We obtained a variation mean value of 7.56 mm, with variation standard deviation of 3.29 mm (Figure 10).

## DISCUSSION

Since the middle of the XX century, with the technological evolution, expert physicians started to have an interest in facial nerve disorders. At this time, there was a development in relation to the diagnoses of the many causes which may involve the nerve, as well as surgical or clinical treatment. Then, there came the need to grade the paralysis, not only to choose the proper treatment method, but also to assess the results obtained, besides the forensic medical records since the first time the patient was seen until the end of the treatment.

Much is discussed about the practicality, reliability and the degree of precision of the methods in order to obtain uniformity between those who analyze a given case or a group of them, in an attempt to cancel the subjectivity of who is analyzing. Nonetheless, in relation to the scientific observation, we need to have an accurate method to quantify this disorder.

The ideal system would provide easy handling, low cost, requiring minimum equipment and little time for its realization, capable of measuring the dynamic and static functions of the facial muscles, and that any examiner could, accurately recognize the real status of the facial paralysis.

Numerous methods and scales have been devised in order to assess, with greater or lesser precision the facial movements and their changes.

In the literature we find subjective methods, which the examiner, based on a scale, assigns paralysis grading as House-Brakmann^28^, Yanagihara^7^ or, even, the system called Toronto Facial Grading System, developed by Ross^10^. Until today, in clinical practice as well as in clinical studies or reports, the method utilized is the one adopted by the Facial Nerve Disorders Committee of the American Academy of Otolaryngology and Head and Neck Surgery, in 1985, created by House & Brackmann^5^.

There were objective methods, from the simplest – such as the Facial Nerve Functional Index, described by Fields and Peckitt^9^, only measured the value between the corner of the mouth and the external corner of the eye, or those which assess the facial movements at rest and in contraction with a simple ruler^14^, ^24^, done directly on the patient, or assessed by means of photographs and films^5^, ^8^.

With the evolution of technology, other, more sophisticated and objective methods were introduced, using cameras, computers and software of varied types^11^, ^12^, ^13^, ^17^, ^18^, ^20^, ^21^, ^22^, ^23^, with the intent of making the method more accurate and cancel subjectivity. It happens that these methods are usually sophisticated and expensive.

In the present study, we used the VICON system, which captures and analyzes human body movements with extreme precision. In 1999, Frey et al.^16^ used this system, which they considered accurate and adequate for scientific purposes; however, not very practical to be utilized in daily practice. We agree with the author, since the equipment is still very costly, it involves special cameras with LED reflectors, costly image capture system and software, besides a proper environment for the test, which makes it difficult, for example, its use in a medical office. It is, however, a method which, because of its accuracy, it can be very useful for doing studies in this field.

Initially, adhesives with 3M reflective tape were used, as many other authors have done^20^, ^22^, ^23^ in predetermined strategic points on the face, to reflect the LED light emitted by the cameras and capture the images. It was necessary to create a facial model in the software to analyze the variation of these points when there was movement.

The system enables evaluation of movement in three dimensions. Nonetheless, we noticed that the movements were linear, basically produced, or mainly produced, in one single plane, changing very little the other planes. For this reason, we analyzed in the present paper, only one of the planes, where there were the greater shift of a given movement, called in the present study as Z plane or axis.

The muscle contraction movements chosen for the analysis were those of smiling, wrinkling the forehead (raising the eyebrows), frowning and closing the eyes. We used the data without differentiating the right and left sides, in order to calculate the movements at rest and after the contraction, considering what can be seen on general tables (attachment), and also according to what some authors have already recorded; these numbers show a statistically irrelevant difference.

In the revised literature, we found detailed methods described, but few define numbers in a normal range so that we can compare the results.

Upon smiling, we obtained a variation between rest and contraction between 6.45 and 12.11mm, which we considered a broad variation, with a mean of 9.28 mm and standard deviation (SD) of 2.83mm. Linstrom et al.^20^, utilized the method called “The Peak Motus Motion Measurement System”, a system which is very similar to what we used, since it used reflexive adhesive points, cameras to capture the images in video and later process them in a software, and they found, for smiling, a mean variation of 5.78 mm, with an SD of 2.09 mm to the right and 5.53 mm with an SD of 1.83 mm to the left. In this case, there is a difference analyzing the mean values between the two studies, which is about 3.3 mm; nonetheless, the aforementioned author does not report on the numbers of variation found upon rest and in contraction and it does not define the number of individuals examined.

As far as the eyes are concerned, we obtained a variation between the open and closed eyelids of 6.89 and 11.29 mm, with a mean of 9.09 mm and SD of 2.20 mm. Again, Linstrom points to a mean value of 8.11 mm with an SD of 2.39 mm to the right and 7.94 mm with an SD of 2.10 mm on the left. Now, the differences found between the two studies were of about 1 mm.

To wrinkle the forehead showed a variation between rest and contraction of 8.06 and 13.08 mm, with a mean value of 10.57 mm and SD of 2.51 mm and frowning had a contraction variation between 4.26 and 10.85 mm, with a mean value of 7.56 mm and SD of 3.29 mm. In the literature we did not find any revised references in order to compare the measures.

It is interesting to observe, as a complement, that the facial muscles contract within a range of approximately 11 and 13 mm, but there is a considerable individual variation for each movement. In this group of young people, in average, for smiling, the movement varied in 5.66 mm from individual to individual; in order to close the eyelids, it varied in 4.4 mm; in order to wrinkle the forehead, it was 5.02 mm and, to frown, it was 6.59 mm.

In facial paralysis cases, it is relevant to document it case by case, thus, the professional will be able to quantify precisely the paralysis development.

## CONCLUSION

This study assessed a new objective method for measuring the contraction of the facial muscles, with the VICON movement capture system, and obtained a normality pattern for the movements of wrinkling the forehead and frowning, smiling and opening and closing the eyes. This is a sophisticated method which, although not feasible for daily clinical use, it can be a good research instrument because of its precision. We observed that the normal facial movements are in the range between 11 and 13 mm of contraction; nonetheless, there is considerable individual variation.
